# Targeting of copper-trafficking chaperones causes gene-specific systemic pathology in *Drosophila melanogaster*: prospective expansion of mutational landscapes that regulate tumor resistance to cisplatin

**DOI:** 10.1242/bio.046961

**Published:** 2019-10-01

**Authors:** Eleni I. Theotoki, Athanassios D. Velentzas, Stamatia A. Katarachia, Nikos C. Papandreou, Nikolas I. Kalavros, Sofia N. Pasadaki, Aikaterini F. Giannopoulou, Panagiotis Giannios, Vassiliki A. Iconomidou, Eumorphia G. Konstantakou, Ema Anastasiadou, Issidora S. Papassideri, Dimitrios J. Stravopodis

**Affiliations:** 1 Section of Cell Biology and Biophysics, Department of Biology, School of Science, National and Kapodistrian University of Athens (NKUA), Athens 15701, Greece; 2 Center of Basic Research, Biomedical Research Foundation of the Academy of Athens (BRFAA), Athens 11527, Greece; 3 Institute for Research in Biomedicine (IRB Barcelona), The Barcelona Institute of Science and Technology (BIST), Barcelona 08028, Spain; 4 Harvard Medical School, Massachusetts General Hospital Cancer Center (MGHCC), Charlestown, Massachusetts (MA) 021004, USA

**Keywords:** Atox1, CCS, Chaperone, Cisplatin, Copper, *Drosophila*, Menkes, Wilson's

## Abstract

Copper, a transition metal, is an essential component for normal growth and development. It acts as a critical co-factor of many enzymes that play key roles in diverse cellular processes. The present study attempts to investigate the regulatory functions decisively controlling copper trafficking during development and aging of the *Drosophila* model system. Hence, through engagement of the GAL4/UAS genetic platform and RNAi technology, we herein examined the *in vivo* significance of *Atox1* and *CCS* genes, products of which pivotally govern cellular copper trafficking in fly tissue pathophysiology. Specifically, we analyzed the systemic effects of their targeted downregulation on the eye, wing, neuronal cell populations and whole-body tissues of the fly. Our results reveal that, in contrast to the eye, suppression of their expression in the wing leads to a notable increase in the percentage of malformed organs observed. Furthermore, we show that *Atox1* or *CCS* gene silencing in either neuronal or whole-body tissues can critically affect the viability and climbing capacity of transgenic flies, while their double-genetic targeting suggests a rather synergistic mode of action of the cognate protein products. Interestingly, pharmacological intervention with the anti-cancer drug cisplatin indicates the major contribution of CCS copper chaperone to cisplatin's cellular trafficking, and presumably to tumor resistance often acquired during chemotherapy. Altogether, it seems that Atox1 and CCS proteins serve as tissue/organ-specific principal regulators of physiological *Drosophila* development and aging, while their tissue-dependent downregulation can provide important insights for *Atox1* and *CCS* potential exploitation as predictive gene biomarkers of cancer-cell chemotherapy responses.

## Abstract (precis)

**Summary:** We demonstrate the essential roles of Atox1 and CCS copper-trafficking chaperones in *Drosophila* development and aging. We also provide insights for their therapeutic exploitation as cisplatin regulators during cancer chemotherapy.

## INTRODUCTION

The maintenance of metal homeostasis in cells is an issue of major importance for an organism's proper function and well-being. Among others, the transition metal copper is a crucial component for survival, growth and development, as it acts as a key co-factor of many enzymes that have major roles in several cellular processes, such as pigmentation of skin, detoxification of reactive oxygen species, mitochondrial electron transport-chain function, as well as procedures related to nerve function, with amidation of neuroendocrine peptides and catecholamine biosynthesis being characteristic examples (Kaler, 2011). However, the redox potential of copper renders it particularly toxic when it is accumulated at high concentrations in the cell (Halliwell and Gutteridge, 1984; Peña et al., 1999; Norgate et al., 2006). This is due to a copper-catalyzed process, known as the Fenton reaction, during which the oxidation of Cu(I) to Cu(II) catalyzes the formation of hydroxyl radicals by hydrogen peroxide (Wardman and Candeias, 1996; Southon et al., 2013).

Copper homeostasis is preserved via the activity of copper transporters involved in metal uptake and export from cells, and copper chaperones that deliver it to specific targets in the cell (Bertinato and L'Abbe, 2004). CTR1 (copper transporter 1) is the major copper import protein (Lee et al., 2002; Denoyer et al., 2015) that belongs to a family of proteins, that is located at the plasma membrane and carries high affinity for copper transport (Dancis et al., 1994; Zhou et al., 2003). CTR1 is responsible for the majority of copper uptake by the cells (Hatori and Lutsenko, 2016). However, DMT1 (divalent metal transporter 1), another copper transporter, is a member of a family of divalent metal transporters and transports metals such as Zn^2+^, Mn^2+^, Cd^2+^, Cu^2+^, Fe^2+^ and others, and also plays a role in copper cellular influx (Gunshin et al., 1997; Au et al., 2008).

Once copper enters the cell, it is trafficked by the three major copper chaperones Atox1, CCS and COX17 and is transferred to the copper-dependent enzymes (Culotta et al., 1999; Zhou et al., 2003). The metal chaperone Atox1 (anti-oxidant 1) is a small cytosolic protein that transfers copper to the secretory pathway, and delivers it to the P-type ATPases ATP7A and ATP7B, located at the *trans*-Golgi network (O'Halloran and Culotta, 2000; Hatori and Lutsenko, 2016; Matson Dzebo et al., 2016). ATP7A releases copper to copper-dependent enzymes in the secretory pathway. When the metal is accumulated at high concentrations in the cell, ATP7A moves to the plasma membrane whereat it promotes copper export from the cell (Balamurugan and Schaffner, 2006). ATP7B is mainly expressed in the liver and kidney, and is responsible for removing copper from the cell through its transport across the plasma membrane (Petrukhin et al., 1994; Balamurugan and Schaffner, 2006; Yu et al., 2017). The CCS (copper chaperone for SOD1) protein delivers copper to SOD1 (superoxide dismutase), an enzyme with a key role in the defense against oxidative stress, which requires the incorporation of zinc and copper ions for its activation (Kirby et al., 2008). Finally, the COX17 (cytochrome *c* oxidase copper chaperone) protein transfers copper to the mitochondrial cytochrome oxidase (Culotta et al., 1999; Zhou et al., 2003).

Because of its great importance as a co-factor for numerous enzymes, absence of copper or deviation from its normal distribution pattern can cause severe developmental abnormalities and baneful diseases. Several neurodegenerative human pathologies, such as Alzheimer's and Parkinson's disease, are likely to be mechanistically associated with copper homeostasis. Most importantly, two typical examples of copper-dependent pathologies are Menkes and Wilson's disease (Adlard and Bush, 2006; Bharathi et al., 2007; Kim et al., 2008). Menkes, an X-linked disorder, is caused by mutations in the *ATP7A* gene, which is required for copper export from the intestinal cells. In Menkes syndrome, the release of copper from the intestinal tract into the blood and its distribution to other tissues are impaired, thus resulting in systemic copper deficiency (Cox and Moore, 2002; van den Berghe and Klomp, 2009; Bahadorani et al., 2010). On the other hand, Wilson's autosomal disorder is caused by mutations in the *ATP7B* gene, which plays an important role in the biliary copper efflux from the hepatocytes. As a result, in Wilson's syndrome, there is an abnormal accumulation of copper in the liver, while its release into the bloodstream leads to serious overload in other organs, particularly the brain and kidney (van den Berghe and Klomp, 2009; Russell et al., 2018). Besides *ATP7A* and *ATP7B*, other genes with aberrant or lack of expression, and/or activity, may detrimentally harm copper homeostasis, and induce tissue pathologies. Hence, their identification would be a novel issue of major clinical and therapeutic importance.

Interestingly, it has been previously shown that copper transporters are also associated with resistance of certain tumors to the anti-cancer drug cisplatin, which is a widely used chemotherapeutic agent against several human malignancies. Cisplatin seems to act by binding to DNA and by activating various signal-transduction pathways, such as the ones involved in DNA-damage recognition, DNA repair, cell-cycle arrest and apoptosis (Siddik, 2003; Kelland, 2007). Nevertheless, some tumor types often develop increased resistance to the drug. In several cases, this has proved to be related to aberrant expression levels of copper transporters. In accordance, it has been shown that the major copper transporter CTR1 plays a key role in the uptake of cisplatin by the cell. In the absence of CTR1, decreased cisplatin concentration in the cell and increased resistance of the cell to the drug are observed (Ishida et al., 2002; Song et al., 2004; Galluzzi et al., 2012). Moreover, oncogenic deregulation of the copper transporters ATP7A and ATP7B also appears to be critically implicated in the control of cisplatin export from the cell, as their overexpression causes reduced drug accumulation in the cancer cell and tumor resistance to the drug (Katano et al., 2002; Siddik, 2003; Song et al., 2004). To this end, we suggest that additional copper homeostasis-regulating proteins may also participate in the cellular trafficking of cisplatin, and, if so, any changes in their expression and/or activity may significantly affect the cisplatin-based chemotherapeutic responses of treated solid tumors.

Therefore, in order to investigate the above raised issues *in vivo*, we herein engaged as a biological platform the *Drosophila melanogaster* model organism, which carries strong and versatile genetic tools. It is widely and successfully used in basic and translational research, and a significant number of its genes are highly homologous to their human counterparts (Reiter et al., 2001; Ong et al., 2015). In the present study, we have examined the *in vivo* role of the two major copper-trafficking chaperones, Atox1 and CCS, during fly development and aging by virtue of their tissue-specific downregulation. Remarkably, the pharmacological intervention with cisplatin has led to clinically relevant conclusions about the potential involvement of these proteins in the cellular trafficking of cisplatin, and, as such, for their future dynamics to be exploited as predictive biomarkers for solid-tumor chemotherapy responses.

## RESULTS

### Copper trafficking that depends on chaperones is an evolutionary conserved process

**Fig. 1.**
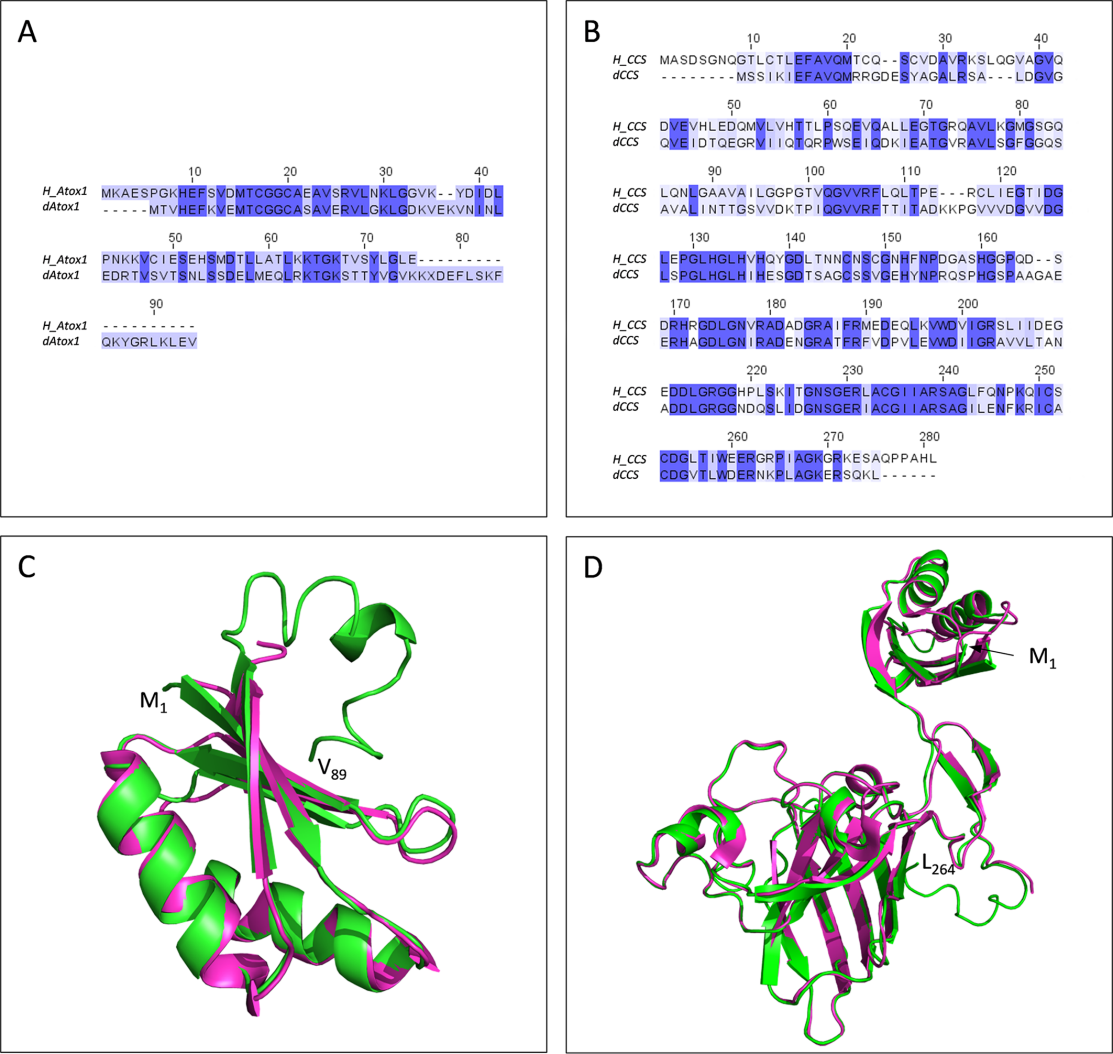
**Strong evolutionary conservation of copper-trafficking chaperones in between fly and human species.** (A) Protein sequence alignment of human Atox1 (*H_ATOX1*) (E5RIM7_HUMAN) with fly Atox1 (*dATOX1*) (M9PD88_DROME) via the Clustal Omega bioinformatics tool. (B) Amino acid sequence alignment in between human CCS (*H_CCS*) (CCS_HUMAN) and fly CCS (*dCCS*) (E1JH26_DROME) proteins via the Clustal Omega sequence alignment program. (C) Structural alignment of *Drosophila* Atox1 (green) with chain A of human Atox1 (magenta), experimentally determined by X-ray crystallography (PDB ID: 3IWX). Alignment was performed using the ‘align’ command of PyMOL software, resulting in an RMSD value of 0.505 Å that clearly states the significant structural similarity between the two examined proteins. (D) Structural alignment of *Drosophila* CCS (green) with chain A of human CCS (magenta). Human CCS was experimentally determined by X-ray crystallography (PDB ID: 6FON). Regarding CCS, alignment was performed utilizing the ‘align’ command of PyMOL software, resulting in an RMSD value of 0.332 Å that corresponds to a strong structural similarity. M, methionine (amino-terminus, 1); V, valine (carboxyl-terminus, 89); L, leucine (carboxyl-terminus, 264).

The Clustal Omega-mediated alignment in between human (*Homo sapiens*) and fly (*D. melanogaster*) Atox1 proteins reveals a strong homology in their amino acid sequences (percentage ID: 48.57), with a remarkable conservation of the ‘MxCxxC’ motif (Fig. 1A), which has been previously reported to serve as a critical site for copper binding (Wernimont et al., 2000; Boal and Rosenzweig, 2009a,b; Matson Dzebo et al., 2016; Kardos et al., 2018). Next, using the same bioinformatics approach (the Clustal Omega tool), human and fly CCS proteins proved to share strong amino acid sequence homologies (percentage ID: 46.07) in between each other, with, however, a striking absence of the ‘MxCxxC’ motif from the *Drosophila* CCS counterpart (Fig. 1B), thereby suggesting the operation of alternative molecular mechanisms for CCS copper binding and its transfer to CCS targets (Schmidt et al., 1999; Stasser et al., 2005, 2007; Kirby et al., 2008; Boal and Rosenzweig, 2009a,b; Fetherolf et al., 2017; Kardos et al., 2018).

To further expand these similarities at the 3D structural level, molecular modeling of fly Atox1 and CCS chaperones was next carried out via employment of the I-TASSER online server, with the quality of predicted models being evaluated via their respective C-scores. C-score is a confidence score that typically ranges from −5 to +2. A high value C-score indicates high confidence in the model. Model predictions were also evaluated utilizing the template modeling-score (TM-score) and root mean-square difference (RMSD) values. TM-score is a scale for measuring the structural similarity in between two proteins with different tertiary structures. A TM-score >+0.5 indicates a model with correct topology, while a TM-score <+0.17 indicates random similarity. The C-score, TM-score and RMSD values for the molecular models of *Drosophila* Atox1 and CCS proteins were calculated as −0.97, 0.59±0.14, 5.7±4.6 Å and 0.84, 0.83±0.08, 4.2±2.8 Å, respectively, thus indicating the tertiary structures' high quality and reliability for both chaperones. To investigate the structural similarity of the predicted models derived from *D. melanogaster* (fly) to the experimentally determined respective structures deposited in Protein Data Bank (PDB) (Berman et al., 2000) from *H. sapiens* (human), the ‘align’ command of PyMOL was applied. ‘Align’ is an automated multi-step superposition algorithm based on dynamic programming and iterative refinement. Initially, it performs a sequence alignment that is followed by a structural superposition and then it carries out zero or more cycles of refinement. In the case of fly Atox1, the derived model was compared to the structure of human Atox1 (Boal and Rosenzweig, 2009a,b) (PDB ID: 3IWX). The calculated RMSD had a value of 0.505 Å, with 61 C_a_ atom pairs having been successfully aligned (Fig. 1C). Similarly, in regard to *Drosophila* CCS, the predicted molecular model was compared to human CCS (Sala et al., 2019) (PDB ID: 6FON), resulting in an RMSD value of 0.332 Å, with 188 C_a_ atom pairs being successfully aligned (Fig. 1D). Altogether, it seems that the chaperone-dependent copper trafficking is a highly conserved process in between human and fly species, and, as such, any genetic and/or pharmacological intervention in a fly setting could be successfully translated into the human pathophysiology and its management in clinical settings.

### Downregulation of the *Atox1* or *CCS* gene does not detectably affect the development of *Drosophila* eye

**Fig. 2.**
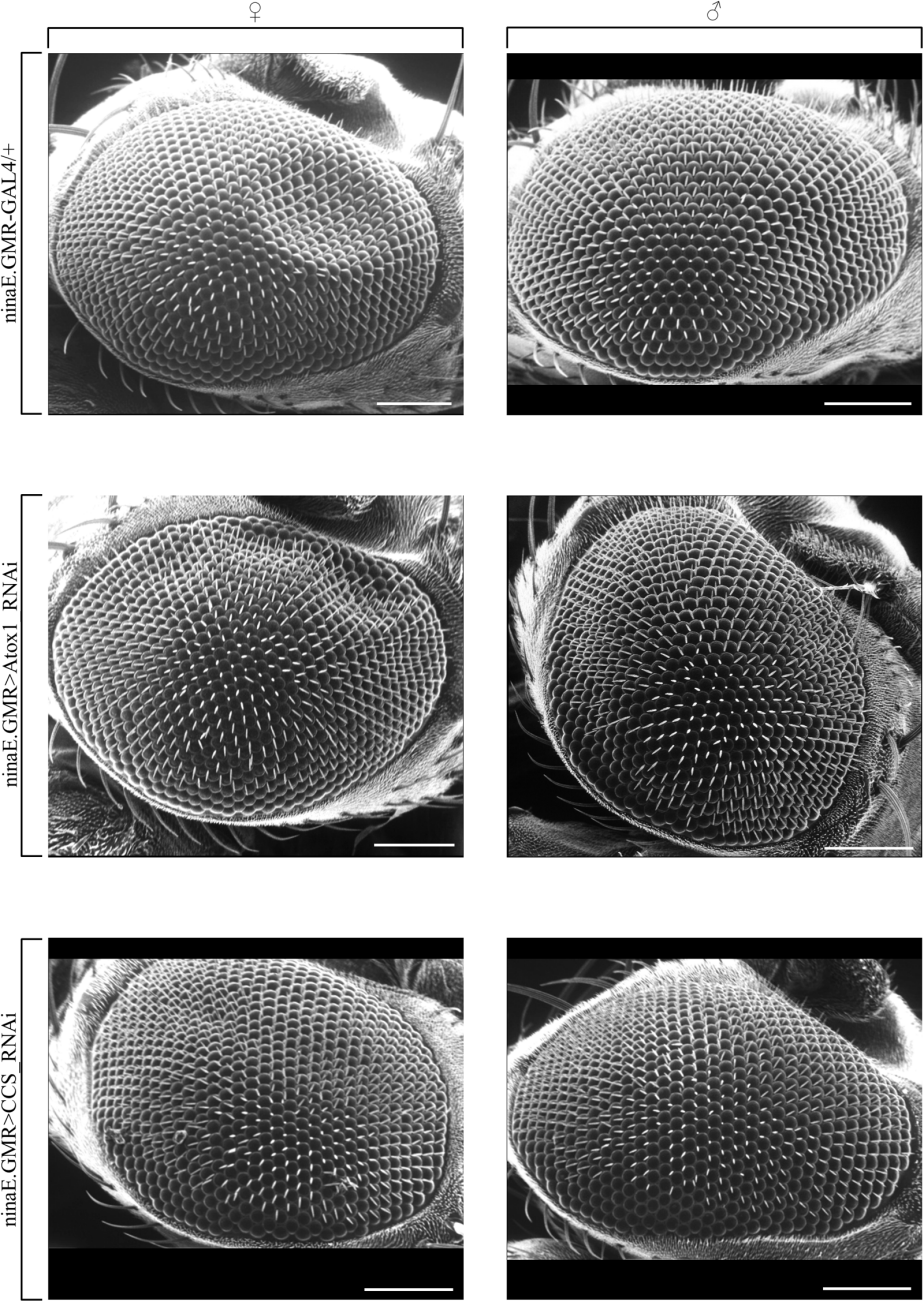
**The dispensable contribution of *Atox1* or *CCS* gene expression to fly eye morphology.** Scanning electron microscopy (SEM) images of compound eyes derived from female (left panels) and male (right panels) transgenic flies at the age of 40 days, carrying downregulated levels of *Atox1* (ninaE.GMR>Atox1_RNAi) (middle panels) or *CCS* (ninaE.GMR>CCS_RNAi) (bottom panels) gene expression, as compared to control (driver-only) conditions (ninaE.GMR-GAL4/+) (top panels). Scale bars: 100 μm.

To determine the role of *D. melanogaster* Atox1 and CCS copper-delivering chaperones, which share significant similarities to their respective human counterparts (Fig. 1) in the development of fly eye during aging, we have downregulated their cognate genes specifically in the (compound) eye, using the GAL4/UAS and RNAi technologies. The two transgenic populations were produced by crossing an eye-specific GAL4 driver-carrying strain with a UAS-Atox1_RNAi or UAS-CCS_RNAi strain, respectively. Next, single-gene-targeted flies (ninaE.GMR>Atox1_RNAi, or ninaE.GMR>CCS_RNAi), at the age of 40 days old, were collected and their eyes were visualized via engagement of a SEM protocol (see Materials and Methods). Control flies (ninaE.GMR-GAL4/+) of the same age were also collected and observed. Our results unveil that neither of the two examined genes seem to play a critical role in the development of *Drosophila* eye, since their eye-specific downregulation seems unable to cause any detectable alteration in organ architectural structure and organization, for both female and male flies (Fig. 2).

### Targeting *Atox1* or *CCS* causes gene-specific increase in the incidents of dysmorphic fly wing formation

**Fig. 3.**
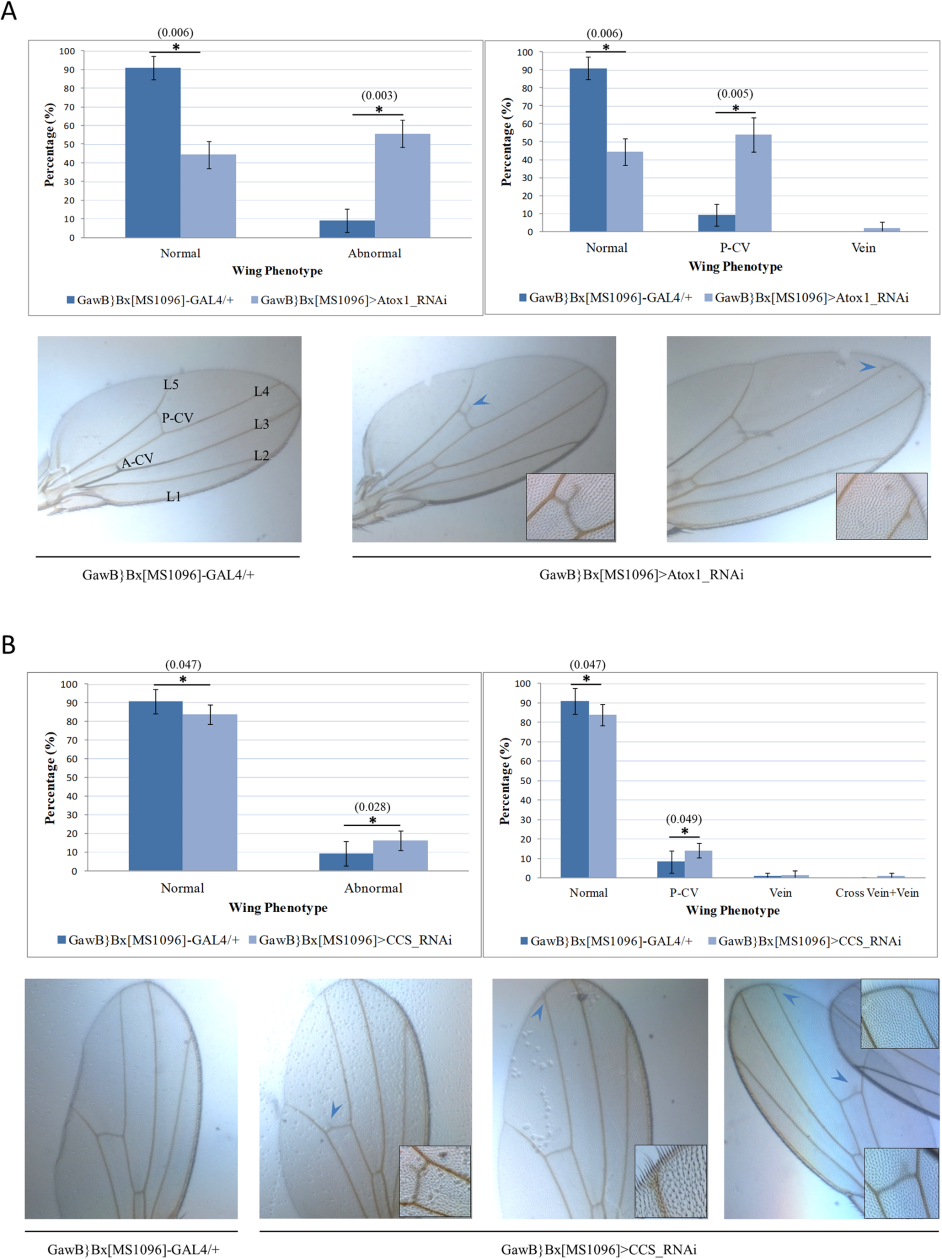
***Atox1* downregulation causes wing pathology in *Drosophila*.** (A,B) Upper panels: bar charts that present the percentage of normal and abnormal (notably dysmorphic) wings (left panels), as well as the percentage of observed vein aberrations, analytically classified in sub-types (right panels), in transgenic flies at the age of 50 days old, which do not express either (A) the *Atox1* gene (GawB}Bx[MS1096]>Atox1_RNAi), or (B) the *CCS* gene (GawB}Bx[MS1096]>CCS_RNAi), as compared to control fly populations (GawB}Bx[MS1096]-GAL4/+). (A,B) Lower panels: light microscopy (LM) images that present typical incidents of vein malformation in pathological wings. Insets: magnifications of wing areas indicated by light blue arrowheads. L1–L5, wing major veins; A-CV, anterior cross-vein; P-CV, posterior cross-vein.

Under physiological conditions, control (GawB}Bx[MS1096]-GAL4/+) fly wings are normally developed and typified by the characteristic morphogenetic pattern of five main veins (L1–L5), a posterior cross-vein (P-CV) and an anterior cross-vein (A-CV) (Fig. 3A). Remarkably, wing-specific-targeted silencing of *Atox1* or *CCS* results in gene-dependent organ pathology. Single-gene-targeted transgenic flies were collected at 50 days old and their wings' structural architecture was examined via a stereomicroscopic approach. Interestingly, *Atox1* downregulation proved to induce a strong dysmorphic effect, since it caused a significant elevation in the percentage of malformed wings developed (Fig. 3A). Specifically, *Atox1*-targeted (GawB}Bx[MS1096]>Atox1_RNAi) flies were presented to carry abnormal wings in a five-times higher percentage than control flies, with the most striking defect being identified in the P-CV structure. On the contrary, regarding CCS, its absence (or reduced levels) does not seem to notably affect wing phenotype as compared to both control and *Atox1*-targeted transgenic flies. CCS loss (GawB}Bx[MS1096]>CCS_RNAi) is unable to produce the extent of alterations in wing morphology observed for the *Atox1*-downregulated flies. Nevertheless, the most pronounced defect was also recognized at the P-CV structure (Fig. 3B).

### Neuronal cell-specific silencing of *Atox1* or *CCS* gene deranges *Drosophila* life expectancy in a sex-dependent manner

**Fig. 4.**
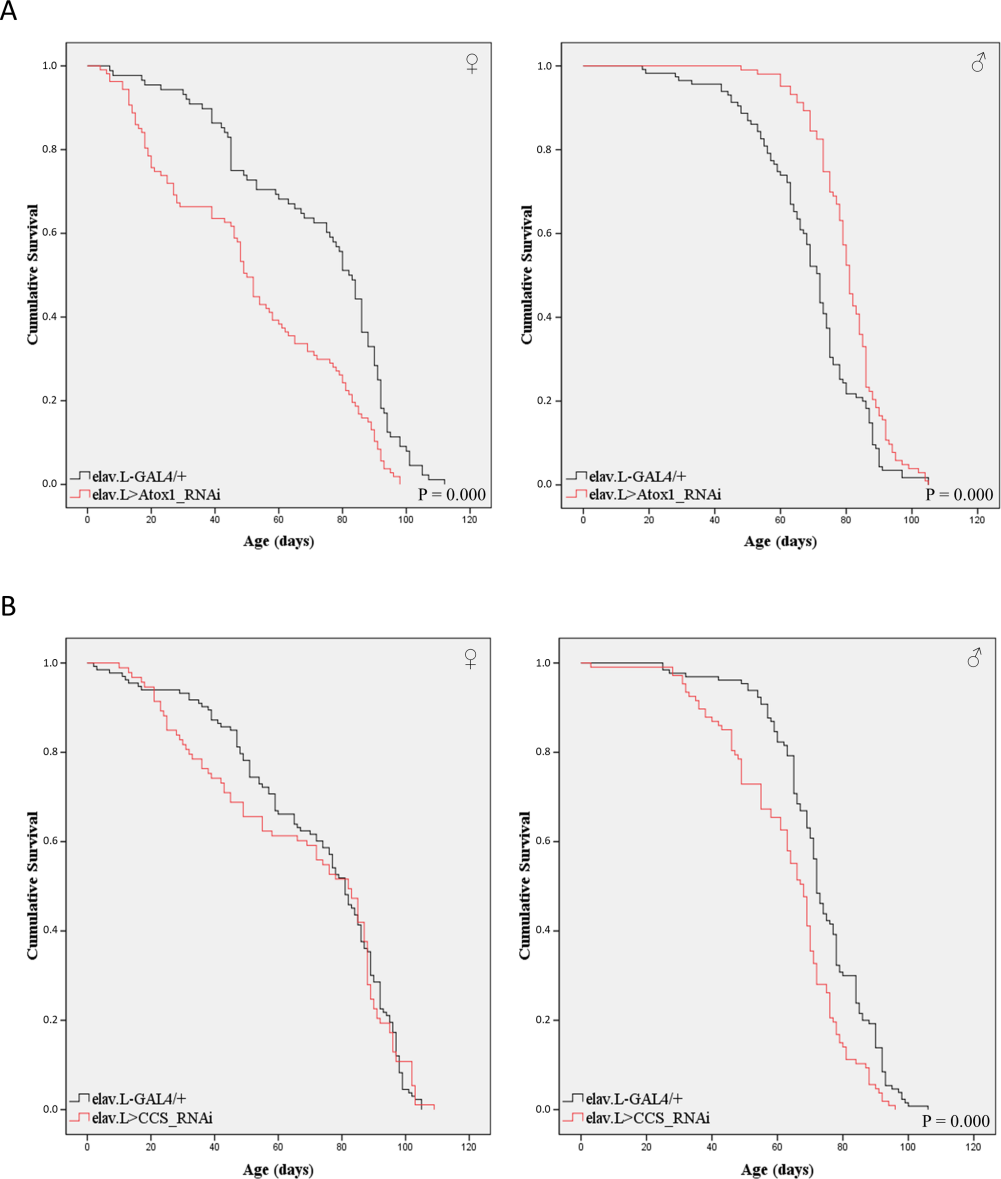
**Neuronal cell-specific targeting of *Atox1* or *CCS* has a sex-dependent impact on fly viability.** (A) Curves presenting the survival rates of transgenic female (left panel) and male (right panel) flies in the absence of *Atox1* gene expression, specifically in neuronal tissues (elav.L>Atox1_RNAi) (red lines), as compared to control fly populations (elav.L-GAL4/+) (black lines). (B) Curves showing the survival rates of female (left panel) and male (right panel) transgenic flies that do not express the *CCS* gene, specifically in neuronal tissues (elav.L>CCS_RNAi) (red lines), as compared to control flies (elav.L-GAL4/+) (black lines).

The importance of the two copper chaperones in the nervous system during aging was examined in transgenic flies carrying downregulated levels of either the *Atox1* or *CCS* gene, specifically in neuronal cells. Female and male individuals were collected and their viability was analyzed on numerous time-points over a lifespan of 120 days. Our results indicate that neuronal loss of *Atox1*, via employment of the elav.L-GAL4 driver (functions specifically in *Drosophila* neuronal cells), significantly alters fly longevity, with the effect strongly depending on the animal's sex (Fig. 4A). Remarkably, *Atox1* gene suppression significantly increases mortality of female (elav.L>Atox1_RNAi), compared to control (elav.L-GAL4/+) flies, whereas the *Atox1*-targeted males show notably elevated viability (Fig. 4A), thus indicating the mechanistic coupling of Atox1-mediated copper trafficking with sex-specific metabolism.

Surprisingly, loss (or reduced levels) of CCS seems unable to prominently impair lifespan in female transgenic flies (elav.L>CCS_RNAi), as their survival profiles do not statistically differ from control (elav.L-GAL4/+), although measurable life expectancy reductions can be observed between the 20th and 80th experimentation day. However, mortality of *CCS*-targeted male flies seems to increase (as compared to control), exhibiting a reverse pattern during aging in comparison to that of *Atox1*-downregulated male flies (Fig. 4B).

### Atox1 and CCS copper chaperones differentially control fly kinetic ability during aging

**Fig. 5.**
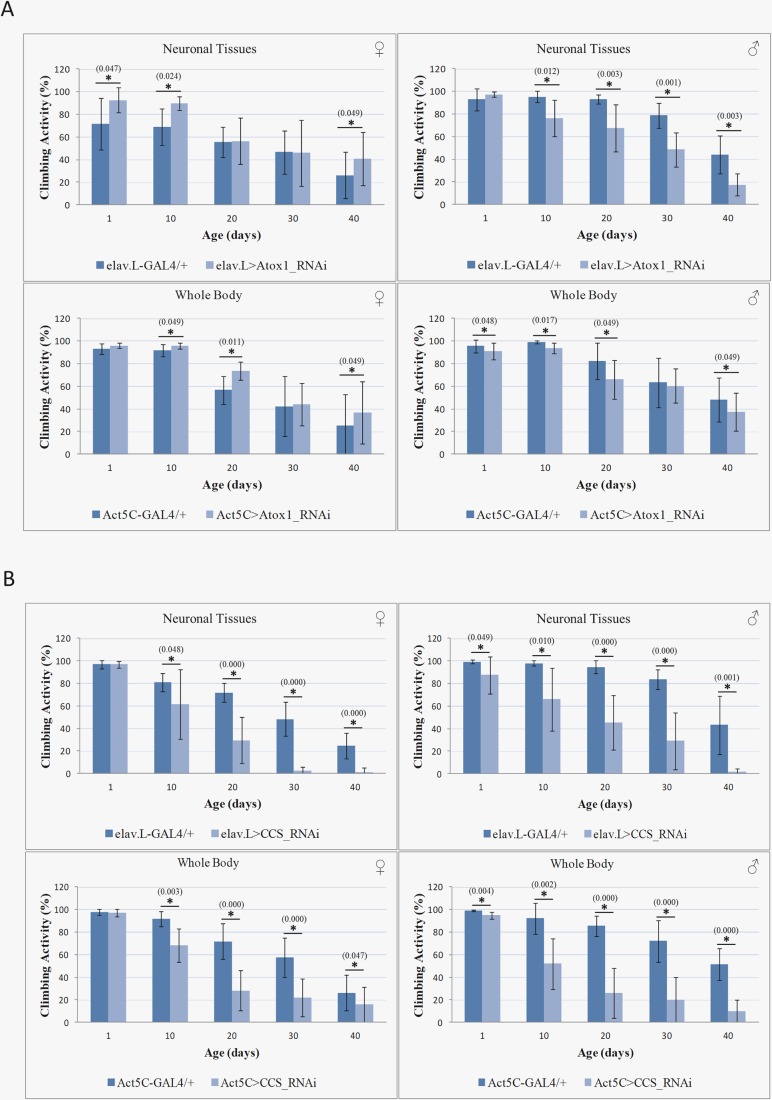
**Lack of *Atox1* or *CCS* expression differentially perturbs kinetic capacity during *Drosophila* aging.** Bar charts showing female (left panels) and male (right panels) transgenic fly climbing activity (%) in the absence of (A) Atox1 (Atox1_RNAi) or (B) CCS (CCS_RNAi) copper chaperone upon respective gene-targeting either in neuronal (elav.L) (upper panels) or whole-body (Act5C) (lower panels) (light blue bars), as compared to control fly populations (elav.L-GAL4/+; Act5C-GAL4/+) (dark blue bars). ‘Climbing activity %’ indicates the percentage of flies who managed to climb over the 60 ml mark of the volumetric cylinder during a period of 20 s, as calculated by the IBM SPSS program (see Material and Methods). Flies at the age of 1, 10, 20, 30 and 40 days old were examined.

Next, neuromuscular systemic integrity was examined in Atox1 and CCS-compromised environments, and compared to normal tissue settings. Flies were subjected to climbing assays and their mobility performances were measured. The obtained data revealed gene-specific and sex-dependent climbing activities, proving that either neuronal cell- or whole-body-specific RNAi-mediated suppression of *Atox1* gene expression differentially affects kinetic proficiencies of female and male fly populations. *Atox1*-targeted females [elav.L>Atox1_RNAi (neuronal tissues) and Act5C>Atox1_RNAi (whole-body)] are inclined to increased mobility, whereas respective males are characterized by reduced climbing activities that follow age-dependent patterns for both (GAL4) genetic drivers used (Fig. 5A). Interestingly, neuronal cell-specific *Atox1* downregulation showed the most significant kinetic pathology on the 40th experimentation day compared to control (elav.L-GAL4/+) flies (Fig. 5A).

Surprisingly, CCS loss resulted in severely pathogenic phenotypes for both sexes compared to Atox1 groups. Targeting the *CCS* gene, either in neuronal or in whole-body tissues of the fly [elav.L>CCS_RNAi (neuronal tissues), or Act5C>CCS_RNAi (whole-body)], caused age-dependent climbing deficiencies, with *CCS* neuronal cell-specific silencing leading to a totally ‘paralytic’ (i.e. complete inability to climb) phenotype for both female and male examined populations on the 40th day of their lifespan (Fig. 5B). Altogether, it seems that CCS, in comparison with Atox1, serves as the principal regulator of the copper-trafficking process during aging of *Drosophila* tissues that are critically engaged in mobility functions.

### Lack of expression of *Atox1* or *CCS* shortens *Drosophila* longevity: gene- and sex-specific profiles

**Fig. 6.**
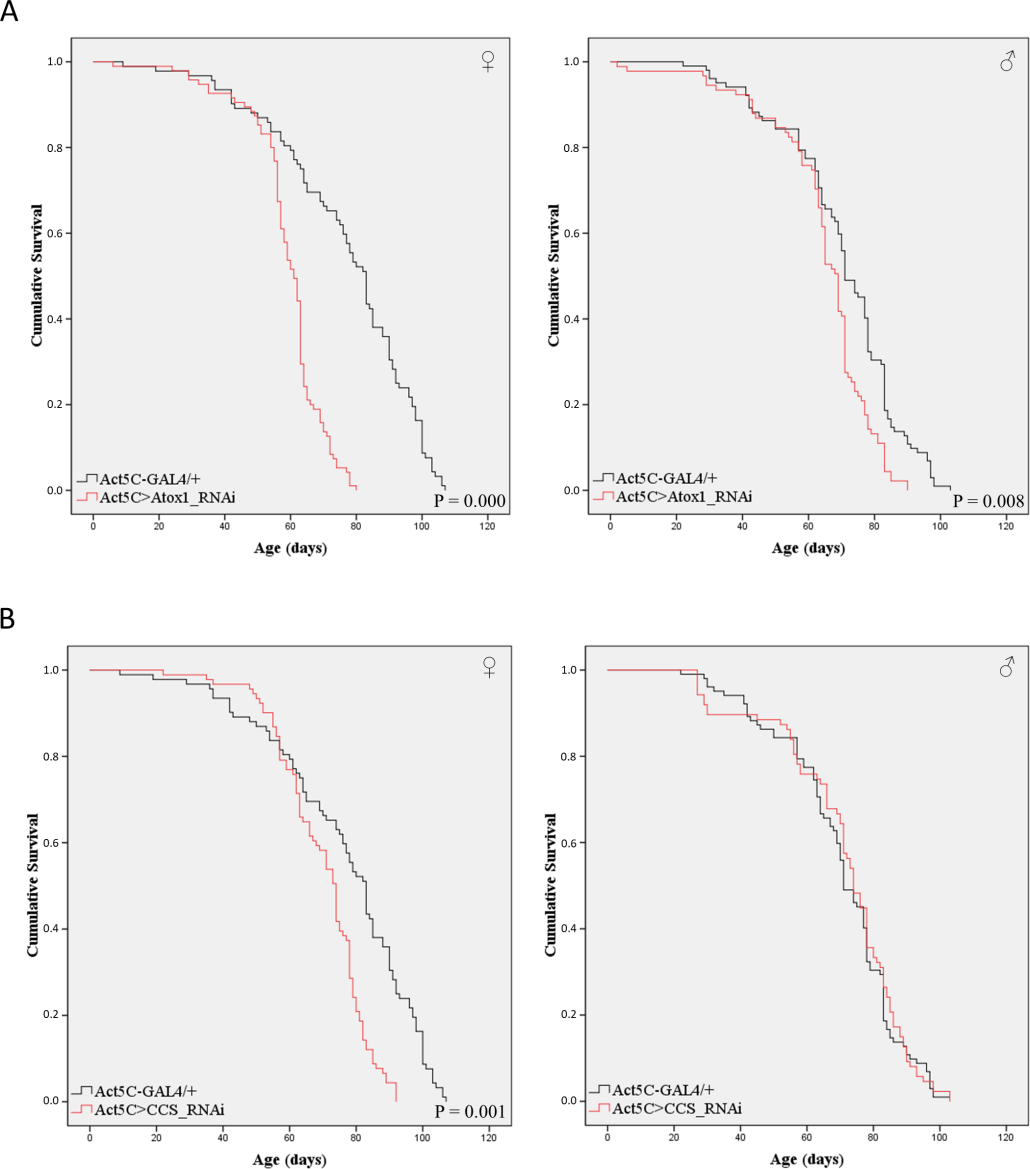
***Atox1* or *CCS* suppression in all organ tissues reduces fly longevity following a sex- and gene-specific pattern.** (A) Curves describing the survival rates of female (left panel) and male (right panel) transgenic flies downregulated for *Atox1* in whole-body *Drosophila* (Act5C>Atox1_RNAi) (red lines), as compared to control fly populations (Act5C-GAL4/+) (black lines). (B) Curves showing the survival rates of female (left panel) and male (right panel) transgenic flies targeted for *CCS* in whole-body tissues (Act5C>CCS_RNAi) (red lines), as compared to control flies (Act5C-GAL4/+) (black lines).

Given the neuronal cell-specific actions of Atox1 and CCS proteins (Figs 4 and 5), we next reasoned that they may play important roles in fly-organ systemic pathophysiology, and especially in lifespan control. Thereby, we downregulated the *Atox1* or *CCS* gene in the *Drosophila* whole-body. Gene-specific and sex-dependent effects in fly survival were detected. Interestingly, Atox1 absence (or reduced expression) [Act5C>Atox1_RNAi (whole-body)], from all body tissues leads to shorter lifespan profiles for both females and males compared to control populations (Act5C-GAL4/+), with female phenotypic mortality significantly higher than in the respective males (Fig. 6A).

Regarding the *CCS* gene, its systemic silencing in all organ tissues [Act5C>CCS_RNAi (whole-body)] causes reduced survival in female but not in male populations (Fig. 6B). Notably, in the context of metal metabolism, Atox1 seems to be more critically implicated in *Drosophila* longevity than the CCS copper chaperone (Fig. 6). However, comparatively opposite gene-targeting pathological patterns are obtained from fly kinetic capacity assays (Fig. 5), thus indicating the *in vivo* reverse relation between mobility-induced energetic stress and long life expectancy in Atox1 or CCS systemically defective environments.

### Double-gene targeting of *Atox1* and *CCS* reveals their functional synergism in the systemic control of *Drosophila* viability

**Fig. 7.**
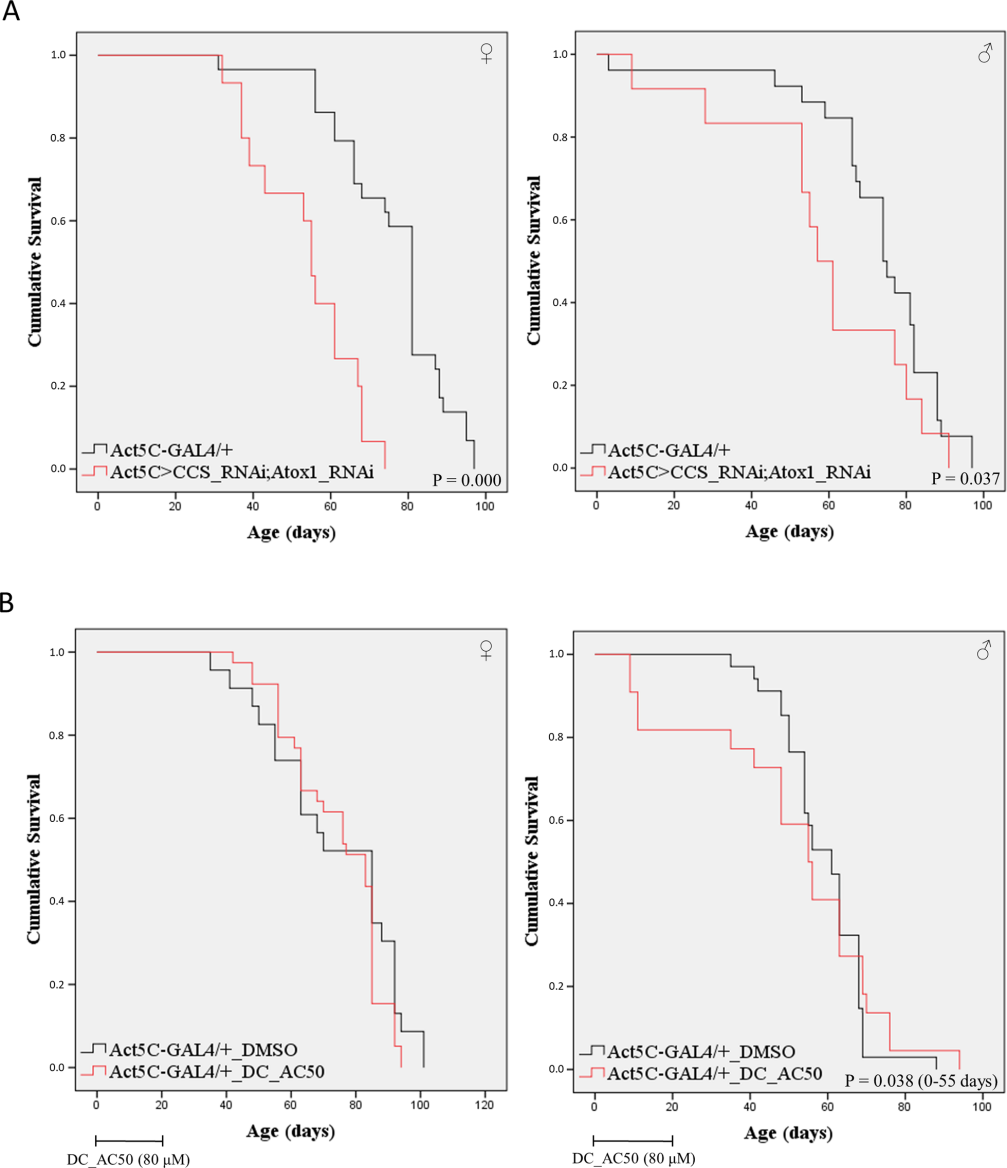
**Double targeting of *Atox1* and *CCS* genes in whole-body tissues induces *Drosophila* shortened viability.** (A) Curves presenting the survival rates of female (left panel) and male (right panel) double-gene-targeted transgenic flies that do not express both *Atox1* and *CCS* genes in whole-body  (Act5C>CCS_RNAi;Atox1_RNAi) (red lines), as compared to control fly populations (Act5C-GAL4/+) (black lines). (B) Survival curves of female (left panel) and male (right panel) control flies (Act5C-GAL4/+) that were subjected to the Atox1- and CCS-chaperone inhibitor DC_AC50 (80 μM) for 20 consecutive days and then maintained in the absence of DC_AC50 until death (red lines). Flies raised with food containing only the DMSO solvent were used as control (black lines).

To investigate *in vivo* if the herein examined genes carry redundant activities or can synergistically contribute to the survival and aging regulation mechanisms, we genetically created double-gene-targeted transgenic flies (Fig. S1), characterized by downregulated expression of both *Atox1* and *CCS* genes in all *Drosophila* organ tissues. Simultaneous silencing of *Atox1* and *CCS* genes [Act5C>CCS_RNAi;Atox1_RNAi (whole-body)] proved to cause more severe systemic mortalities (Fig. 7A) compared to the single-gene (*Atox1* or *CCS*)-targeting-induced ones (Fig. 6) for both fly sexes, with females subjected to more tissue detriments and survival losses than males (Fig. 7A). Remarkably, the viabilities of double-gene-silenced transgenic populations decrease faster and more steeply (Fig. 7A) compared to the those observed in *Atox1* or *CCS* single-gene whole-body-specific suppression (Fig. 6).

Besides their genetic downregulation, we attempted to pharmacologically target *Drosophila* copper chaperones by using the small organic molecule DC_AC50, a human Atox1 and CCS inhibitor (Wang et al., 2015). Control (Act5C-GAL4/+) flies were exposed to either DMSO (group of reference) or DC_AC50 (80 μM) for 20 days and then allowed to survive in the absence of the inhibitor. In contrast to females, male populations underwent a notable lifespan reduction in response to DC_AC50, especially during the first 50 days of age (Fig. 7B). This sex-specific DC_AC50 action is likely to be associated with the female fly's ability to efficiently metabolize and detoxify the applied inhibitor *in vivo*. On the other hand, DC_AC50 capacity to partly ‘phenocopy’ (Fig. 7B) the double-gene (*Atox1* and *CCS*)-targeting-induced *Drosophila* enhanced mortality (Fig. 7A) in male flies, strongly suggesting the critical (and presumably sex hormone-dependent) *in vivo* roles of Atox1 and CCS copper-trafficking chaperones in the survival and aging of all biological organisms, including invertebrates (fly) and mammals (human).

### CCS copper chaperone serves as a major regulator of cisplatin trafficking and cytotoxicity in *Drosophila* tissues during aging

The widely clinically administered anti-cancer drug cisplatin (cDDP) utilizes copper transporters for its cellular influx and efflux (Kelland, 2007; Kuo et al., 2007). CTR1 is involved in cisplatin uptake by the cell, whereas ATP7A and ATP7B seem to control its export from the cell (Song et al., 2004; Kelland, 2007; Kuo et al., 2007). Loss-of-function mutations in these genes can modify tumor-cell response to the drug (Kelland, 2007). Hence, we have reasoned that other copper-homeostasis regulators, such as the Atox1 and CCS chaperones, could also critically contribute to cisplatin-induced toxicity and tissue harm. This is strongly supported by the property of human Atox1 to bind cisplatin (Boal and Rosenzweig, 2009a,b; Palm et al., 2011; Palm-Espling and Wittung-Stafshede, 2012). Alterations in *Atox1* and/or *CCS* gene expression may decisively deregulate cisplatin's trafficking routes and interactions with target molecules (i.e. nuclear DNA), thereby reshaping cellular phenotypes of drug resistance.

**Fig. 8.**
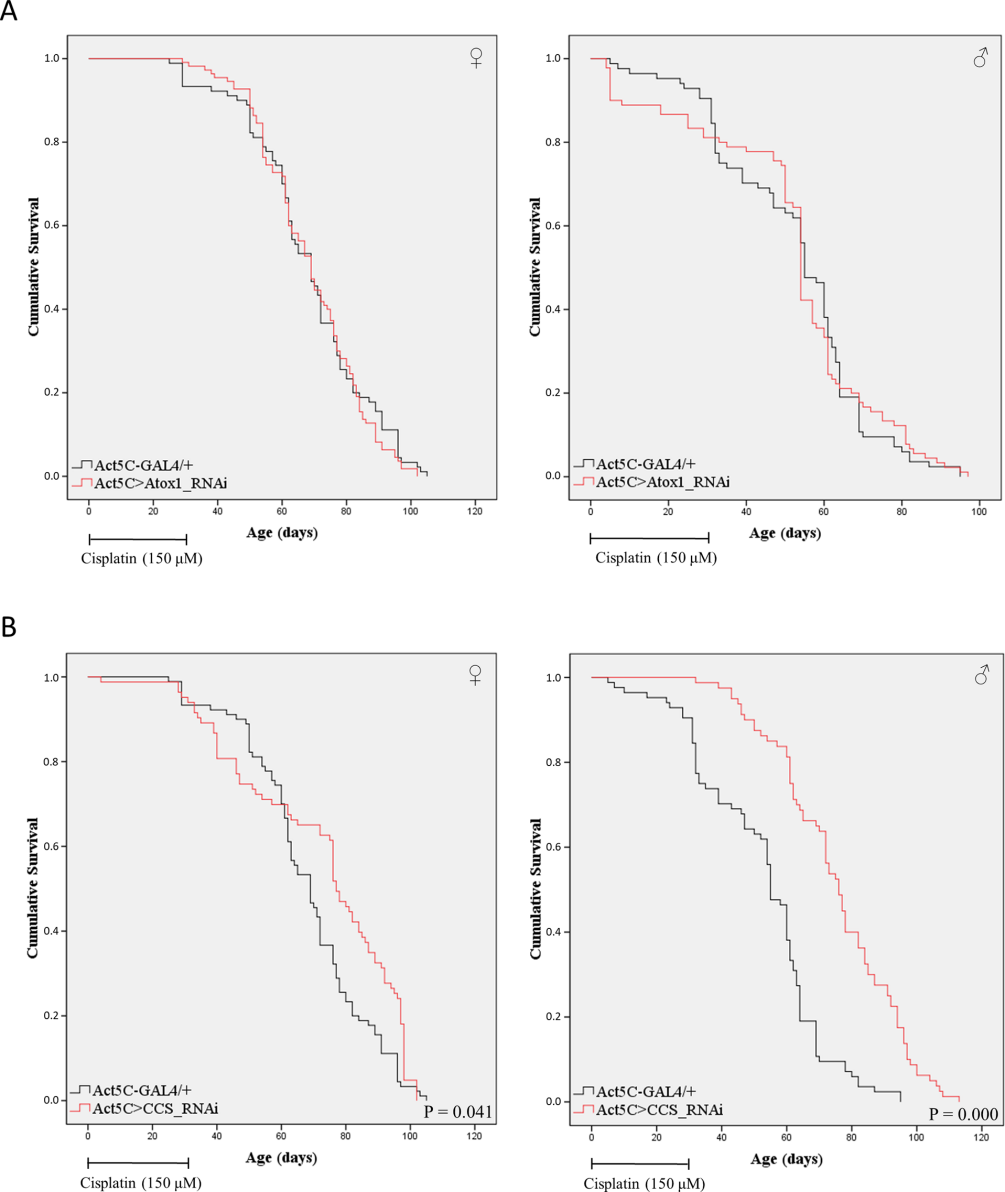
**Lack of *CCS*, but not *Atox1*, expression desensitizes *Drosophila* cells from cisplatin toxicity.** (A) Survival rates of female (left panel) and male (right panel) *Atox1*-targeted flies (Act5C>Atox1_RNAi) that were subjected to cisplatin (150 μM) exposure (supplemented into the food) for 30 consecutive days (red lines), as compared to cisplatin-treated control (Act5C-GAL4/+) populations (black lines). (B) Survival curves of female (left panel) and male (right panel) *CCS*-targeted flies (Act5C>CCS_RNAi) that were exposed to cisplatin (150 μM) (supplemented into the food) for 30 consecutive days (red lines), with Act5C-GAL4/+ serving, in the presence of cisplatin, as control (black lines). After the first 30 days, flies were allowed to live in the absence of cisplatin until death.

To investigate this scenario, we have herein examined how fly viability is affected by the pharmacological intervention with cisplatin *in vivo*. Flies were exposed to cisplatin (150 μM; administered in the food) for 30 consecutive days, and then allowed to survive and age in the absence of the drug. Our results reveal that cisplatin causes a significant elevation of mortality rates in control (Act5C-GAL4/+) flies (Fig. 8; compare to Fig. 6), with a reduction level of approximately 15 and 26% for females and males, respectively, observed at the 50% survival rate of the populations. Remarkably, the detrimental effects of cisplatin in the survival of transgenic flies with downregulated *Atox1* or *CCS* expression follow gene-specific patterns during aging. In contrast to *Atox1* [Act5C>Atox1_RNAi (whole-body)] (Fig. 8A), it is the *CCS* gene systemic silencing [Act5C>CCS_RNAi (whole-body)] that renders *Drosophila* organ tissues partly refractory to cisplatin's cytotoxic activity, thereby resulting in notably improved viability profiles (Fig. 8B). The ability of *CCS*-targeted male flies to more efficiently tolerate and/or neutralize the cell-killing actions of cisplatin, as compared to the female respective populations (with a measurable survival benefit observed only after the 60th experimentation day) (Fig. 8B), could likely be associated with a sex hormone-dependent expression/regulation/activity of CCS protein (Fig. 6B) and/or detoxification pathways of cisplatin. Altogether, it seems that cisplatin intracellular trafficking, and thus cytotoxicity, in the fly is mainly controlled by the CCS copper chaperone. Nevertheless, a role of CCS activity in functionally compensating for the lack of Atox1 cannot be excluded, thus indicating the Atox1 effective involvement in cisplatin metabolism. Interestingly, it may be the CCS and/or Atox1 absence (or downregulation, or mutation-driven loss of function) that is critically implicated in drug resistance mechanisms, being frequently observed during cancer chemotherapy.

### TCGA-derived mutational and expression profiling of genes encoding the Atox1 and CCS copper chaperones may dictate cisplatin resistance during human cancer therapy

**Fig. 9.**
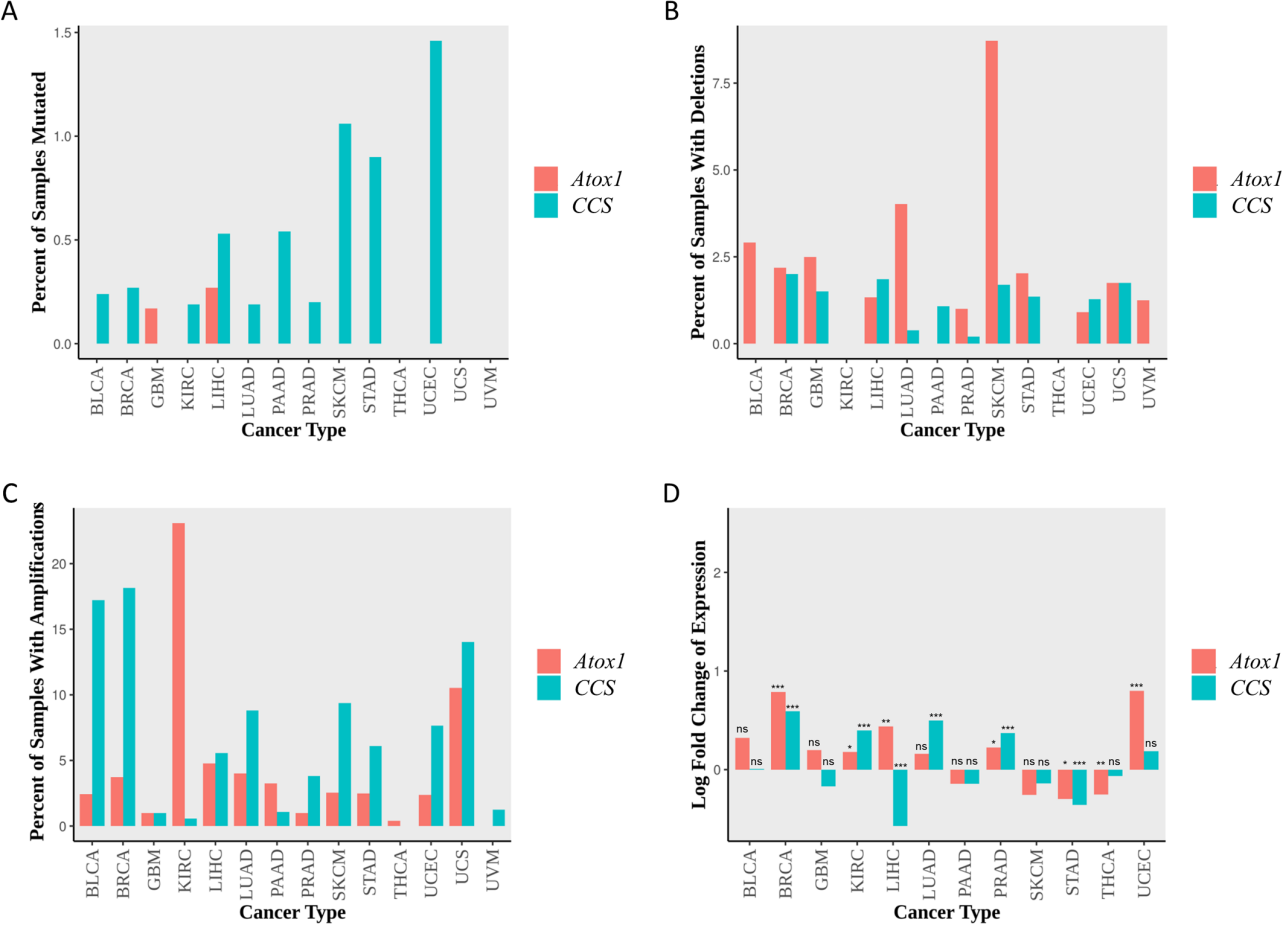
**TCGA-derived mutational and expression landscaping of *Atox1* and *CCS* in representative human cancers.** *Atox1* and *CCS* alterations are collectively described in 14 human cancer types derived from the TCGA platform. The two genes are color-coded; coral for *Atox1* and blue for *CCS* in all plots. (A) Percentage of samples mutated in each of the 14 cancers examined. The mutation types considered were: translation start-site mutations, splice-site mutations, non-stop mutations, nonsense mutations, missense mutations and indels (regardless of in-frame or frame-shifting mutations). (B,C) Percentage of samples carrying somatic copy-number deletions (B) or copy-number gains (amplifications) (C). GISTIC2 data are thresholded by intensity to ±1 and ±2, with ±1 indicating gain or loss of one copy number, and therefore one allele, and ±2 indicating deep amplification or deletion. Amplifications or deletions are much longer in size than indels shown in the mutation bar chart. (D) Log-2 fold changes of (*Atox1* or *CCS*) gene activities in cancer versus normal samples for each of the 12 human malignancies analyzed. No differential expression analysis could be performed for UCS and UVM, since there were no normal samples studied in either dataset. All *P*-values were adjusted using the Benjamini-Hochberg method. *P<0.05, **P<0.01, ***P<0.001; ns, not significant. BLCA, bladder cancer; BRCA, breast cancer; GBM, glioblastoma multiforme; KIRC, kidney renal-cell carcinoma; LIHC, liver hepatocellular carcinoma; LUAD, lung adenocarcinoma; PAAD, pancreatic adenocarcinoma; PRAD, prostate adenocarcinoma; SKCM, skin cutaneous melanoma; STAD, stomach adenocarcinoma; THCA, thyroid carcinoma; UCEC, uterine corpus endometrial carcinoma; UCS, uterine carcinosarcoma; UVM, uveal melanoma.

The Cancer Genome Atlas (TCGA) is a publicly funded project by the National Cancer Institute (NCI) and The National Institutes of Health (NIH). It has provided the cancer-research community with an unprecedented deluge of data derived from more than 30 different cancers and spanning over 11,000 samples (Tomczak et al., 2015; Hutter and Zenklusen, 2018). To examine the potential contribution of *Atox1* and *CCS* genes to cisplatin resistance of human tumors, the available multi-omics data of TCGA platform for 14 distinct cancer types were thoroughly analyzed. It seems that neither the *Atox1* nor *CCS* gene are often mutated in any of the cancers examined, with the highest mutation frequency for *CCS* and *Atox1* observed in uterine corpus endometrial carcinoma (UCEC) (1.5%) and liver hepatocellular carcinoma (LIHC) (0.27%) malignancies, respectively (Fig. 9A). In terms of somatic copy-number alterations, *Atox1* is subjected to a deletion process in a high percentage of skin cutaneous melanoma (SKCM) samples (8.7%), while the deletion percentage, for either gene, could not exceed the 5% value for all the remaining 13 cancers studied (Fig. 9B). Nevertheless, both genes follow cancer-specific patterns of amplification, with *Atox1* and *CCS* being amplified at a maximal level in kidney renal-cell carcinoma (KIRC) (23.1%) and breast cancer (BRCA) (18.1%) diseases, respectively (Fig. 9C). Strikingly, regarding their differential expression in 12 out of the 14 cancers analyzed, neither *Atox1* nor *CCS* genes are found significantly upregulated, with the comparably strongest expression levels being respectively measured in UCEC and BRCA malignancies. Furthermore, 6 out of 12 (50%) cancers are characterized by detectably downregulated *Atox1* and/or *CCS* gene activities (Fig. 9D).

Altogether, it seems that tumor cells mobilize diverse mechanisms to impair copper homeostasis and cisplatin metabolism. Providing that Atox1 and CCS copper chaperones are essentially engaged in trafficking of cisplatin and in its targeting to specific molecules, their cognate gene mutations, deletions or downregulations may render tumor cells refractory to cisplatin-based therapy. Remarkably, all 12 different cancers examined were shown to carry low levels of *Atox1* and *CCS* gene expression (Fig. 9D), thereby suggesting their potential ability to develop resistance against cisplatin during malignancy clinical management. Hence, before applying a cisplatin-dependent regimen to a cancer patient, the thorough mutational and expression profiling of (among others) *Atox1* and *CCS* genes are strongly advised.

## DISCUSSION

*D. melanogaster* serves as a powerful, reliable and versatile model organism for the *in vivo* study of animal development and growth. In this work, we have employed state-of-the-art genetic tools of the fly, such as the GAL4/UAS (Duffy, 2002) and RNAi (Tomari and Zamore, 2005) technologies to investigate the contribution of the major copper-trafficking chaperones Atox1 and CCS to development and aging.

Surprisingly, upon silencing of *Atox1* or *CCS* gene expression in the compound eye, no detectable dysmorphia could be observed (Fig. 2), indicating that neither *Atox1* nor *CCS* play a significant role in fly eye development during aging. In contrast, lack of Atox1 seems to cause a notable pathology in wing vein morphogenesis, with the percentage of abnormal wings exceeding 50% (Fig. 3). Since *CCS* targeting is associated with weak wing pathology (Fig. 3), it must be Atox1, but not CCS, activity that critically controls the transport of copper to the secretory pathway and its subsequent delivery to copper-dependent enzymes (Boal and Rosenzweig, 2009a,b; Hatori and Lutsenko, 2016; Matson Dzebo et al., 2016; Kardos et al., 2018) in *Drosophila* wing tissues.

Regarding the downregulation of *Atox1* or *CCS* gene expression specifically in neuronal tissues, both viability and mobility were significantly affected, following sex- and gene-dependent patterns. In the absence of Atox1, fly female mortality is increased, whereas in males it is reduced (Fig. 4), thus suggesting the crucial contribution of sex hormone-mediated metabolism to Atox1 neuronal functions. Remarkably, neuronal cell-specific loss of Atox1 leads to moderately elevated mobility (climbing activity) in females and notably diminished mobility in males (Fig. 5). Interestingly, it seems that *Atox1*-targeted (in neuronal cells) flies carrying increased kinetic abilities are also characterized by reduced longevities and vice versa. This likely indicates that the elevated mobility caused by Atox1 loss in neuronal tissues results in the fast exhaustion of the targeted flies, quickly driving them to energy-depletion-induced death. On the contrary, slower movement can lead to longer life expectancy, presumably due to enhanced energy preservation and limited accumulation of toxic metabolites. Atox1 protein transfers copper primarily to ATP7A and ATP7B ATPases, for its subsequent delivery to target proteins being critically implicated in diverse cellular processes (O'Halloran and Culotta, 2000; Matson Dzebo et al., 2016; Kardos et al., 2018). Hence, if certain enzymes require copper to control neuropeptide production, or other nervous system-related mechanisms (Kaler, 2011), Atox1-depletion must significantly impair fly kinetic capacity and lifespan in a neuropeptide deficiency-dependent manner. Strikingly, copper ‘dyshomeostasis’ has been previously accepted as a hallmark of several neurodegenerative diseases, including Alzheimer's and Parkinson’s (Bush, 2013; Davies et al., 2016; Greenough et al., 2016; James et al., 2017; Kardos et al., 2018).

Remarkably, silencing of the *CCS* gene, specifically in neuronal cells, causes dramatic reduction of kinetic activity (mobility) for both *Drosophila* sexes during aging (Fig. 5) and a notable compromise in male fly longevity (Fig. 4). Our results clearly unveil the essential role of CCS copper chaperone in neuronal cell-dependent climbing of aged flies *in vivo*, which directly reflects the major contribution of the copper-trafficking process to neuro-muscular integrity and potency in elderly individuals. Interestingly, since SOD1 and XIAP (X-linked inhibitor of apoptosis) proteins represent two of the well-established CCS targets, it may be their critical de-regulation in neuronal cells that drives flies to ‘paralytic’ phenotypes and lifespan pathologies in *CCS*-targeted settings. CCS chaperone transfers copper to SOD1 enzyme, the maturation and activation of which secure cellular defense against oxidative stress (Wong et al., 2000; Lamb et al., 2001; Boal and Rosenzweig, 2009a,b; Banci et al., 2012; Matson Dzebo et al., 2016). We suggest that the presumably defective function of SOD1, in the absence of CCS, leads to a significant increase of oxidative load and stress in neuronal tissues, thus directing the development of ‘paralysis’ and shortening of longevity. Besides SOD1, CCS is also capable of delivering copper to XIAP, causing its inactivation and therefore promoting apoptosis (Mufti et al., 2006; Brady et al., 2010; Matson Dzebo et al., 2016). Upon CCS downregulation, copper likely cannot be delivered to XIAP, freeing its ability to suppress apoptosis. This XIAP anti-apoptotic activity may counterbalance the impaired SOD1-induced oxidative cell damage, ultimately lowering CCS lack-driven mortality. Involvement of an X-linked factor, such as the XIAP protein in human species, could differentially control animal sex responses in the absence of the CCS chaperone. However, the *Drosophila* XIAP homologs, DIAP1 and DIAP2, are not located on the X chromosome, hence indicating the implication of another novel X-linked CCS target or regulator that can critically contribute to the sex-specific pathology observed in flies missing the CCS copper-trafficking function in neuronal tissues. Obviously, sex-dependent metabolic and/or hormonal networks must decisively orchestrate the distinct pathogenic phenotypes being developed in *CCS*-silenced male and female environments (Figs 4 and 5).

Comparison of the *CCS*-targeted *Drosophila* climbing-activity-profiles to the *Atox1*-targeted profiles during aging either in neuronal or whole-body tissues (Fig. 5) indicates the genes’ differential contribution to fly kinetic ability, with CCS serving as the major chaperone controlling animal mobility. However, a role of CCS in functionally (even partly) compensating for the absence of Atox1 cannot be ignored. Interestingly, a lack of Atox1 in all tissues of the body generates age-dependent kinetic pathologies with similar patterns to neuronal cell-specific ones (Fig. 5). This strongly suggests that among body tissues it is the neuronal cell compartments (including brain) that critically contribute to the movement disabilities of *Atox1*-downregulated flies. It seems that the ability of Atox1 to mediate copper trafficking is subjected to sex-specific regulation in most fly tissues, including neuronal ones (Figs 4–6), with hormonal and/or metabolic male networks significantly differing from the female networks. Intriguingly, Atox1 has an additional property to act as copper-dependent transcription factor that controls *CCND1* (*cyclin D1*) gene expression, the protein product of which is essentially involved in cell division, promoting the transition from G1- to S-phase of the cell cycle (Itoh et al., 2008; Matson Dzebo et al., 2016). Furthermore, by employing yeast two-hybrid screening technology, several new interaction partners for Atox1 have been identified, including, besides ATP7A and ATP7B, the DNMT1 (DNA methyl-transferase 1) protein, which propagates methylation patterns to the next generation (Ohrvik and Wittung-Stafshede, 2015; Matson Dzebo et al., 2016). Hence, in *Drosophila*, absence of Atox1 may cause methylation-mediated reprogramming of gene expression and/or CCND1-dependent cell-cycle arrest, ultimately inducing tissue dysfunction, kinetic impairment and longevity compromise.

To further examine the potential redundancy or functional synergism between the two copper chaperones, we have simultaneously targeted both *Atox1* and *CCS* genes in all tissues of the *Drosophila* body. *Atox1* and *CCS* double-gene silencing revealed the synergistic mode of action of their cognate proteins, since the lifespan pathology in these flies is more severe compared to single-gene-targeted flies (Figs 6 and 7). It must be both Atox1- and CCS-target de-regulations that produce the obtained pathogenic phenotypes. Double-gene genetic targeting and double-protein pharmacological (DC_AC50; Wang et al., 2015) inhibition undoubtedly demonstrate the importance of chaperone-mediated copper trafficking in animal survival and kinesis *in vivo* (Fig. 7).

The pathology generated by suppression of *Atox1* or *CCS* gene in *Drosophila* neuronal tissues can serve as a powerful and reliable model system for Menkes and Wilson's diseases in human. Nevertheless, previous reports, besides the *ATP7A-* and *ATP7B*-responsible genes, failed to identify pathogenic mutations in the *Atox1* gene in a number of patients (Moore et al., 2002; Kralik et al., 2017). This indicates, among others (i.e. promoter- or intron-residing alterations), an *Atox1* mutation-directed lethality, or an *Atox1* mutation-diminished frequency in the affected populations. Alternatively, it could be the *CCS*, but not *Atox1*, gene mutations that are mechanistically and causatively associated with Menkes and/or Wilson's pathogenic phenotypes. Apart from disease-related mutations, downregulation of *Atox1* and/or *CCS* gene expression, in either all or specific body (i.e. neuronal) tissues, might also result in the development of Menkes- or Wilson's-like symptoms. Importantly, in contrast to previous studies (Kirby et al., 2008; Hua et al., 2011), our genetic strategy allows not only whole-body- but also tissue-specific- (i.e. neuronal) targeting of *Atox1* or *CCS* gene, in both single-gene- and double-gene-silencing schemes. Through engagement of selected GAL4 drivers, ‘kinetic mapping’ of fly brain compartments and cell sub-populations that carry genetically defective *Atox1* or/and *CCS* gene activities will likely prove beneficial for the mechanistic illumination and successful clinical management (i.e. prognosis, diagnosis and therapy) of copper-trafficking diseases, including Menkes and Wilson’s.

Given the previously published reports examining the effects of Atox1 loss on cisplatin pharmacology in diverse cellular settings (Safaei et al., 2009; Hua et al., 2011; Palm et al., 2011; Bompiani et al., 2016), we have herein investigated the *in vivo* roles of Atox1 and CCS copper-trafficking chaperones in cisplatin systemic toxicity. Surprisingly, *CCS*, but not *Atox1*, downregulation proved capable of significantly decreasing cisplatin-induced mortality in *Drosophila* (Fig. 8). Based on the molecular modeling of fly Atox1 protein (Fig. 1), its carboxyl-terminal tail amino acid sequence may structurally mask, or functionally hinder, the closely located ‘RKTGK’ pentapeptide sequence (Fig. 1) to likely act as a nuclear localization signal (NLS), similar to that proposed for the human Atox1 homolog (Matson Dzebo et al., 2016). If so, *Drosophila* Atox1 cannot enter the nucleus to ensure genome integrity from cisplatin mutagenic activity. On the other hand, CCS copper chaperone is herein presented to serve as a critical regulator of cisplatin toxicity in the fly model system (Fig. 8), presumably operating as a drug transporter/carrier to its cognate targets (i.e. nuclear DNA). Depending on tissue settings and genetic contents, Atox1 and CCS chaperones can control cisplatin trafficking routes and targeting efficiencies, thus indicating their essential contribution to acquired resistance of tumor cells to cisplatin-containing therapeutic regimens. Indeed, several types of different human cancers analyzed via employment of the TCGA bioinformatics platform are characterized by strikingly diminished levels of *Atox1* and *CCS* gene expression compared to control (Fig. 9). Providing the tissue-specific decisive implication of Atox1 and CCS chaperones in cisplatin trafficking, targeting and toxicity, tumors profiled with significantly downregulated Atox1 and CCS protein contents must be prone to developing cisplatin resistance. Therefore, cancer patients must be appropriately advised on the importance and necessity of mutational and expression profiling of genes controlling copper/cisplatin homeostasis and metabolism before entering cisplatin-based therapeutic protocols and trials in clinical settings.

In conclusion, our results demonstrate the essential roles of Atox1 and CCS copper-trafficking chaperones in *Drosophila* development and aging *in vivo*, while they also provide valuable insights for the prognostic, diagnostic and therapeutic exploitation of Atox1 and CCS copper/cisplatin regulators during cancer chemotherapy and clinical management of the disease.

## MATERIALS AND METHODS

### Protein molecular modeling

The 3D predictions for Atox1 and CCS fly copper-trafficking chaperones were generated using the I-TASSER (Iterative Threading ASSEmbly Refinement) online server (Roy et al., 2010; Yang et al., 2015) for automated protein structure and function prediction, without changing the default parameters of the software. Structural models of the obtained protein sequences were produced via multiple-threading alignments and iterative structural-assembly simulations. Comparison of the constructed models with structures of other known proteins can provide insights for the function of each examined protein (Yang et al., 2013). Sequences of Atox1 and CCS *Drosophila* protein homologs were retrieved from UniProt (2019) (respective accession numbers: M9PD88 and E1JH26). Images illustrating structural models were prepared by the PyMOL molecular visualization system (http://www.pymol.org).

### Fly stock maintenance

Flies were raised on fly food, which contained 500 ml water, 4 g agar, 3.9 g dry yeast, 16 g sugar, 32 g rice-flour, 25 g tomato paste, 2 ml ethanol and 2 ml propionic acid. Fly stocks were maintained at 25°C.

### Construction of single-gene-targeted transgenic flies

Targeted downregulation of *D. melanogaster Atox1* and *CCS* genes was achieved using the GAL4/UAS genetic system (Duffy, 2002) and RNAi technology (Tomari and Zamore, 2005). Single-gene-silenced transgenic *D. melanogaster* populations were created after crossing flies expressing a tissue-specific driver (GAL4) with ones carrying an RNAi construct being controlled by the presence of a UAS element. The tissue-specific drivers used were: w[*]; P{w[+mC]=GAL4-ninaE.GMR}(Kuo et al., 2007)12 (BL: 1104), W[1118] P{w[+mW.hs]=GawB}Bx[MS1096] (BL: 8860), w[*]; P{w[+mC]=GAL4-elav.L}3 (BL: 8760) and y[1] w[*]; P{w[+mC]=Act5C-GAL4}25FO1/CyO, y[+] (BL: 4414), all obtained from the Bloomington *Drosophila* Stock Center (NIH P40OD018537) (Indianapolis, USA). The transgenic *D. melanogaster* UAS-Atox1_RNAi (ID: 23057) and UAS-CCS_RNAi (ID: 108665) strains were provided by the Vienna *Drosophila* Resource Center (Vienna, Austria). Each (GAL4) driver's tissue specificity (promoter activity) was tested using GFP (green fluorescent protein) as reporter protein (data not shown).

### Construction of double-gene-targeted transgenic flies

Double-gene-downregulated transgenic flies were generated using a fly strain carrying suitable markers and balancers (if/cyo;sb/tm6b) (Fig. S1). We crossed if/cyo;sb/tm6b with UAS-Atox1_RNAi and UAS-CCS_RNAi, separately, and after a number of selected crossings, a double-gene-targeted transgenic fly strain for *Atox1* and *CCS* copper chaperones (CCS_RNAi/CCS_RNAi;Atox1_RNAi/Atox1_RNAi) was produced.

### Light microscopy (LM)

*D. melanogaster* wings were visualized using a Nikon microscope, model Digital Eclipse C1 (Nikon; Tokyo, Japan). Flies were allowed to grow for 50 days and were then mass-collected. After their micro-dissection, wings were placed onto microscopic slides and observed via LM technology.

### Scanning electron microscopy (SEM)

To examine the structural architecture of *D. melanogaster* compound eye, we used a high-definition Phillips 515 scanning electron microscope. Female and male transgenic flies, at the age of 40 days, were collected and immediately sacrificed by an ice-chilling process. Next, flies were air-dried at room temperature, attached to aluminum stubs and coated with gold-palladium (60–40%). Observation and imaging were carried out through SEM technology.

### Survival assay

To study viability, approximately 200 flies (∼100 from each sex) at the age of 0–3 days old were collected and anesthetized by exposure to diethyl ether. The female and male populations were separated from each other and placed in separate vials (20–25 individuals/vial). Deceased flies were measured every day until death. Vials were kept in a constant temperature and humidity chamber throughout the whole experimentation period, and flies were given fresh fly food every 5–6 days.

### Climbing assay

Flies at the age of 0–3 days old were collected, anesthetized (by exposure to diethyl ether), and females were separated from males before the test. Flies were left in quiescence for about 2 h to recover from narcosis. Next, they were placed in a 100 ml volumetric cylinder and left to climb. The number of flies who managed to climb over the 60 ml mark of the volumetric cylinder during a period of 20 s was carefully counted and the whole process was repeated six times for each sample. After assay completion, female and male individuals were kept together again to avoid induction of stress. Climbing assays were repeated for flies at the age of 10, 20, 30 and 40 days old.

### Double pharmacological targeting with DC_AC50

To pharmacologically target both Atox1 and CCS copper chaperones, the synthetic small molecule DC_AC50 (Sigma-Aldrich, Missouri, USA), an Atox1 and CCS inhibitor (Wang et al., 2015), was employed, using DMSO as its suitable solvent. Flies were allowed to survive and grow on fly food containing 80 μM of DC_AC50 for 20 days and were subjected to a survival assay as previously described. Control flies, which developed and grew on fly food containing 80 μM DMSO, were also examined. Flies were given fresh fly food every 6 days. After 20 days of exposure, fly viability continued to be measured in the absence of the inhibitor.

### Pharmacological intervention with cisplatin

The anti-cancer drug cisplatin was added into fly food at a final concentration of 150 μM, and fly viability was measured as previously described. Flies were given fresh fly food every 3 days. After 30 days of exposure to cisplatin, flies kept growing in the absence of the drug.

### Gene profiling

Briefly, mutation and indel data, generated by seven different variant calling algorithms, were obtained by using the TCGAmutations package (Ellrott et al., 2018). Next, somatic copy-number alteration data, produced through employment of the GISTIC2 algorithm (Mermel et al., 2011) and raw RNA-Seq count data, generated via the HT-Seq framework (Anders et al., 2015),⁠ were obtained using the TCGAbiolinks package (Colaprico et al., 2016). Copy-number data quantify gains or losses of chromosomal parts, which may or may not contain genes (Staaf et al., 2008). Differential gene-expression analysis was performed for 12 cancers [UCS (uterine carcinosarcoma) and UVM (uveal melanoma) cancer samples lack normal counterparts], after implementing TMM (Trimmed Mean of M-values) normalization (Robinson and Oshlack, 2010) and transforming the normalized read counts using the Voom tool (Law et al., 2014). Finally, data were fitted to a linear model and weighted using the empirical Bayes procedure (Ritchie et al., 2015). The *P*-values were adjusted for multiple comparisons using the Benjamini-Hochberg method (false discovery rate) (Benjamini and Hochberg, 1995) and the significance level was set at 0.05. Results are presented in Log-2 values of each respective fold change. Notably, for a few cancer types, such as skin cutaneous melanoma (SKCM; 1), pancreatic adenocarcinoma (PAAD; 4) and glioblastoma multiforme (GBM; 5), there are only a small number of normal samples that have hitherto been examined, which could partly compromise the statistical power of differential gene-expression analysis.

### Statistical analysis

All statistical analyses (except gene profiling) were conducted using IBM SPSS version 22. For the climbing assay and wing morphology, the results were presented as mean±SSD (sample standard deviation), and significance was evaluated using one-way ANOVA. For the survival assay, the Kaplan–Meier statistical test was employed. For all tests, statistical significance was accepted at *P*<0.05.

## Supplementary Material

Supplementary information
