# Supplementary material for: Targeting of copper-trafficking chaperones causes gene-specific systemic pathology in Drosophila melanogaster: prospective expansion of mutational landscapes that regulate tumor resistance to cisplatin
Source: Biol Open. 2019 Oct 1;8(10):bio046961. doi: 10.1242/bio.046961 (PMC6826294; doi:10.1242/bio.046961)
Supplement: Supplementary information [file biolopen-8-046961-s1.pdf]

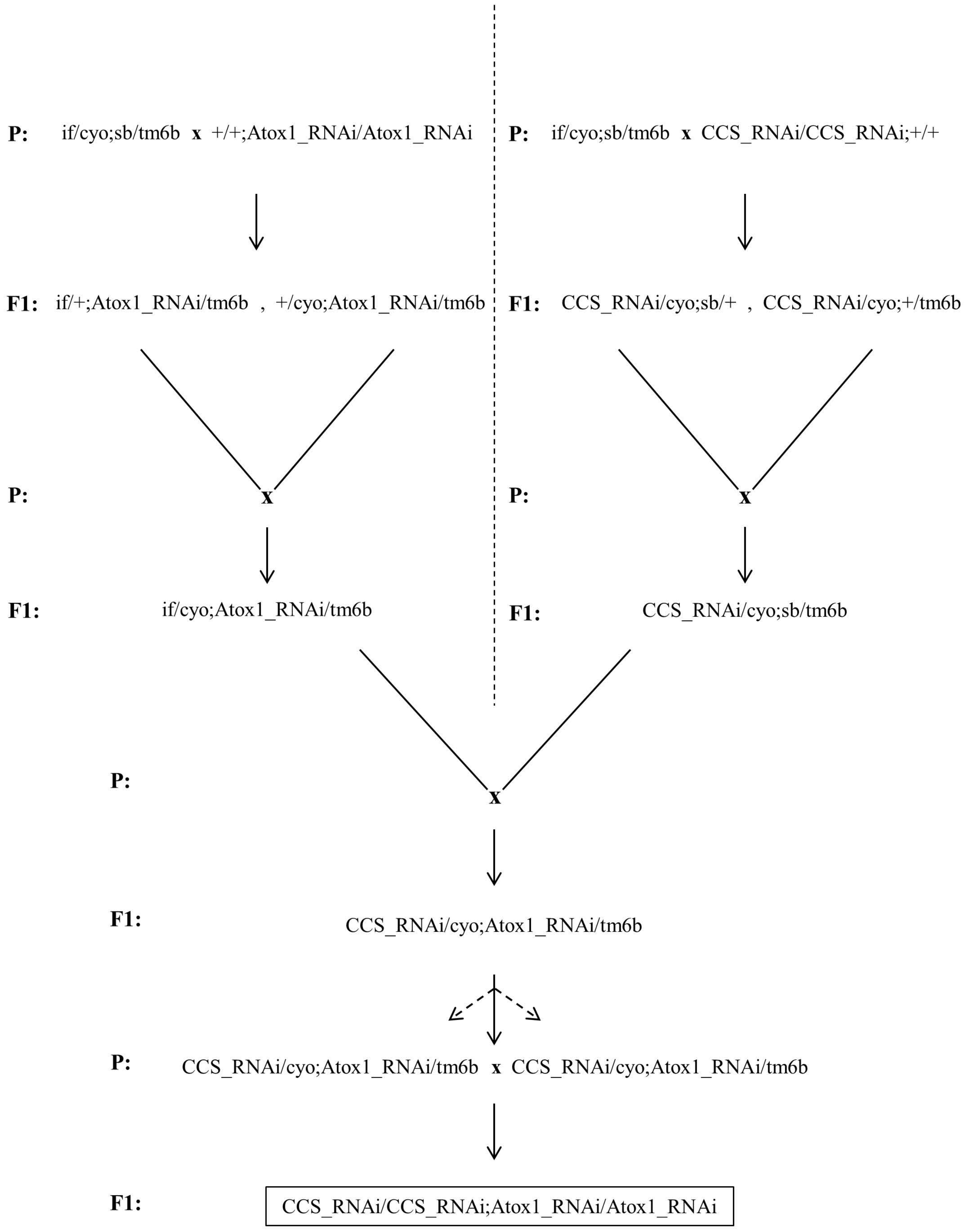

Figure S1. Construction of double-gene targeted transgenic flies. Diagram that presents the series of selected crossings for production of the double-gene targeted transgenic fly strain CCS_RNAi/CCS_RNAi;Atox1_RNAi/Atox1_RNAi. A fly strain carrying suitable markers and balancers (if/cyo;sb/tm6b) was used in this genetic protocol.

Biology Open • Supplementary information
